# Construct Validity and Reliability of the Arabic Version of Hypertension Knowledge-Level Scale Among Saudi Population

**DOI:** 10.7759/cureus.33182

**Published:** 2022-12-31

**Authors:** Sulaiman A Alshammari, Almaha H Alshathri, Sarah S Aldharman, Aljohara H Alshathri, Jana K Abukhlaled, Durrah W Alabdullah, Sarah Aleban

**Affiliations:** 1 Family and Community Medicine, King Saud University, Riyadh, SAU; 2 College of Medicine, King Saud University, Riyadh, SAU; 3 College of Medicine, King Saud Bin Abdulaziz University for Health Sciences, Riyadh, SAU; 4 College of Medicine, Princess Nourah Bint Abdulrahman University, Riyadh, SAU

**Keywords:** saudi, arabic, hypertension knowledge-level scale, reliability, validity

## Abstract

Background

Knowledgeable people are more likely to follow their treatment plan and reduce hypertension morbidity and mortality. This study aimed to construct the validity and reliability of the Arabic version of the Hypertension Knowledge-Level Scale (HK-LS) among Saudis.

Methods

This cross-sectional questionnaire research targets hypertensives and non-hypertensives. Cronbach's alpha and Spearman's correlation matrix analysis measured the questionnaire's construct validity. Wilcoxon rank sum tests examined HK-LS tools' discriminant validity. A p<0.05 was considered significant.

Results

A total of 1424 responses were received. However, the 1419 individuals were evaluated after applying exclusion criteria. About 60% of the participants were female, with the most common age groups being 18-29 years (38.5%) and 40-49 years (24.0%). A personal history of hypertension was reported by 26.1%, and a family history of hypertension by 73.3% of the subjects. Cronbach's alpha for the whole HK-LS was 0.883, suggesting that the tool was reliable. All the items of the HK-LS questionnaire were significantly correlated with each other, except for a non-significant correlation between statements two and seven (r=0.05, p=0.091). The strongest correlations were apparent between items 19 and 20 (r=0.70, p<0.0001), items one and two (r=0.64, p<0.0001), and items 18 and 19 (r=0.56, p<0.0001). However, the weakest associations were reported among items two and six (r=0.06, p<0.05), items two and nine (r=0.07, p<0.05), and items one and 19 (r=0.07, p<0.05). The discriminant validity showed that a personal history of hypertension was associated with significantly higher scores of two HK-LS subscales, including definitions and complications. In addition, the overall knowledge score was significantly higher among participants with a positive history of hypertension among family and/or friends.

Conclusions

The Arabic version of the HK-LS was found to be a reliable and valid tool for measuring knowledge about hypertension among the Saudi population. This reliable instrument can assist medical professionals in establishing education programs.

## Introduction

Knowledge is acquiring information and skills via instruction and experience [1]. Its assessment in the health field is crucial since it may assist professionals in coordinating care and educational initiatives. Measuring an individual's knowledge of chronic conditions like systemic arterial hypertension (SAH) impacts treatment adherence [2]. The patient's education, cooperation, and active involvement in the treatment process are essential since systemic arterial hypertension is a quiet and aggressive condition.

It is well recognized that knowledgeable individuals are more likely to positively modify how they care for their health [3,4]. Furthermore, a lack of understanding and inaccurate perceptions about hypertension impact and limit one's quality of life [2]. Therefore, Turkish researchers designed the Hypertension Knowledge-Level Scale (HK-LS) to assess the knowledge level about SAH of individuals over 18. In 2012, the original version was published in English and is widely used worldwide [5]. In 2015, the HK-LS was utilized in Iran to assess important factors in hypertension knowledge [6]. Also, in 2016, it was translated into Arabic, cross-culturally modified, and used to evaluate Jordanian adults' knowledge of SAH [1]. Additionally, the instrument was translated into Greek [7], and research demonstrating its use in the Polish version was published in Poland [8].

Several studies translate and validate the scale (HK-LS) in different languages [5,7-11]. For example, it was translated and validated into the Indonesian version [9]. Moreover, two studies in Brazil were conducted. The first study was conducted in 2018 by Arthur et al. for translation and cross-cultural adaptation of (HK-LS) [10]. In 2021, the same authors, Arthur et al., published the Brazilian version, testing the validity and reliability of (HK-LS) [11]. In 2019, in Pakistan, the researchers used the scale to ascertain the knowledge of hypertensive patients [12]. The HK-LS was originally developed in Turkish and English but has been translated and cross-culturally adapted into Greek, Polish, Indonesian, Brazilian, and Arabic [5,7-11].

The Arabic version was only translated and cross-culturally adapted. The validation of the HK-LS in the Saudi culture makes the valid and reliable instrument available for measuring adult knowledge of hypertension that has been utilized in other contexts. Given the above, the Arabic version of the scale had its content translated and cross-culturally adapted; however, it was not evaluated as to the other psychometric properties as validity and reliability in the Arabic population, a fact that led to the development of this research, which has the following guiding question: "Is the Arabic version of the Hypertension Knowledge-Level Scale reliable and does it evaluate the knowledge of people with hypertension about the disease?". On these bases, the authors aimed to assess the construct validity and reliability of the Arabic version of the Hypertension Knowledge-Level Scale (HK-LS) among Saudis.

## Materials and methods

This descriptive cross-sectional questionnaire-based study was conducted in Saudi Arabia between August 2022 and December 2022. The target subjects were the general population of Saudi Arabia from different areas (Western, Central, Eastern, Southern, and North). The study was conducted through a self-administered questionnaire. The authors distributed and collected the questionnaire online from different regions of Saudi Arabia and exported it into a Microsoft Excel (Microsoft Corporation, Redmond, Washington, USA) spreadsheet file using Google Docs tools for processing and analyzing information. Data were analyzed using RStudio (R version 4.1.1., Posit, Boston, MA, USA). 

Sample size and sampling technique

Using OpenEpi® version 3.0 (Centers for Disease Control and Prevention (CDC), Atlanta, GA, USA), the sample size required was 385, with a margin of error of 5%, a confidence level of 95%, and a population size of 34 million, which is the Saudi population. We adjusted the estimated sample size to 400 participants to overcome incomplete questionnaires. This study aimed to recruit at least 220 participants based on the guidelines for the respondent-to-item ratio ranging from 10:1 (220 respondents for a 22-item questionnaire) [13]. 

Inclusion criteria and exclusion criteria

The study's eligibility criteria include both hypertensive and non-hypertensive individuals aged ≥18 years in Saudi Arabia. Those individuals who declined to participate in the study and did not fill out the whole questionnaire were excluded from this research.

Data collection instrument and process

The author of the previous study in Jordan provided us with the questionnaire's Arabic translation and permission to use it [1]. The questionnaire consists of two main sections. The first covers sociodemographic characteristics such as age, gender, education level, employment, and personal and family history of hypertension. The second section contains the HK-LS items. The HK-LS consisted of 22 items to investigate participants' knowledge regarding six subscales, including definition (two items), medical treatment (four items), drug compliance (four items), lifestyle (five items), diet (two items), and complications (five items). Participants' knowledge was based on summing up the correct responses to these items (detailed in Appendix 1, Table 6). Therefore, the overall knowledge score ranged between 0 and 22. 

All information was confidential, and participation in this study was voluntary, with the participants' informed consent being obtained on the first page before filling out the questionnaire. Furthermore, ethical approval was obtained from the Institutional Review Board Committee at King Saud University before initiating the study (reference No. 22/0690/IRB). 

Statistical analysis

The authors used RStudio (R version 4.1.1., Posit, Boston, MA, USA) for data analysis. They presented categorical variables as frequencies and percentages, while numerical variables were presented as mean ± standard deviation (SD). We assessed the questionnaire's construct validity by quantifying the internal consistency (using Cronbach's alpha) and conducting Spearman's correlation matrix analysis. In addition, the researchers evaluated the discriminant validity of the HK-LS tools by investigating the differences in knowledge scores based on the personal and familial history of hypertension using a Wilcoxon rank sum test. A p<0.05 was considered statistically significant.

## Results

Demographic characteristics

We received the responses of 1424 participants on the online platform, of whom five respondents declined to participate. Therefore, the data of 1419 participants were analyzed. More than half of the participants were female (60.1%) and had obtained a university degree (64.9%), whereas employed respondents represented 45.5% of the sample. The most frequent age groups were 18-29 years (38.5%) and 40-49 years (24.0%). A personal history of hypertension was reported among 26.1%, a positive family history was apparent among 73.3%. Details of the demographic characteristics of participants are shown in Table 1.

**Table 1:** Demographic characteristics.

Parameter	Category	N (%)
Gender	Male	566 (39.9%)
	Female	853 (60.1%)
Age	18-29	547 (38.5%)
	30-39	206 (14.5%)
	40-49	340 (24.0%)
	50-59	269 (19.0%)
	60 or more	57 (4.0%)
Educational level	General education	409 (28.8%)
	University	921 (64.9%)
	Post-graduate	89 (6.3%)
Employed	Yes	645 (45.5%)
Personal history of hypertension	Yes	370 (26.1%)
History of hypertension among family/friends	Yes	1040 (73.3%)

Results of the internal consistency analysis

The reliability analysis of the HK-LS subscales showed that Cronbach’s alpha coefficient was the lowest for the medical treatment and drug compliance subscales (Cronbach’s alpha=0.597 and 0.708, respectively). The highest internal consistency indicators were reported for the complications subscale (Cronbach’s alpha=0.846) and the definition subscale (Cronbach’s alpha=0.789). Details of reliability statistics and a description of knowledge scores of the HK-LS questionnaire are shown in Table 2.

**Table 2:** Reliability statistics and description of knowledge scores of the HK-LS questionnaire. HK-LS: Hypertension Knowledge-Level Scale, SD: standard deviation.

Domain/subscale	Number of items	Cronbach’s alpha coefficient	Knowledge score	
			Mean ± SD	Min-max
Definition	2	0.789	1.02 ± 0.85	0-2
Medical treatment	4	0.597	2.65 ± 1.22	0-4
Drug compliance	4	0.708	2.47 ± 1.32	0-4
Lifestyle	5	0.750	3.74 ± 1.46	0-5
Diet	2	0.723	1.08 ± 0.91	0-2
Complications	5	0.846	3.80 ± 1.61	0-5
Overall HK-LS	22	0.883	14.75 ± 5.01	0-22

The Cronbach’s alpha of the overall HK-LS was 0.883, indicating a reliable survey tool. The removal of two statements (each at once) resulted in a higher Cronbach’s alpha coefficient. These items included item 1 (Cronbach’s alpha=0.885) and item 2 (Cronbach’s alpha=0.885). Table 3 shows the results of the internal consistency of the HK-LS questionnaire with the removal of each item at once.

**Table 3:** Results of the internal consistency of the HK-LS questionnaire with the removal of each item at once. HK-LS: Hypertension Knowledge-Level Scale, SD: standard deviation.

Item	Cronbach’s alpha	Mean ± SD
Overall	0.883	14.75 ± 5.01
HKLS_01	0.885	14.95 ± 8.28
HKLS_02	0.885	15.13 ± 8.27
HKLS_03	0.880	15.59 ± 8.30
HKLS_04	0.881	14.94 ± 8.39
HKLS_05	0.881	15.26 ± 8.25
HKLS_06	0.879	15.02 ± 8.30
HKLS_07	0.878	14.95 ± 8.32
HKLS_08	0.878	14.87 ± 8.34
HKLS_09	0.880	15.05 ± 8.25
HKLS_10	0.879	14.94 ± 8.33
HKLS_11	0.879	14.81 ± 8.35
HKLS_12	0.878	15.43 ± 8.22
HKLS_13	0.877	15.53 ± 8.20
HKLS_14	0.878	14.86 ± 8.33
HKLS_15	0.876	15.58 ± 8.19
HKLS_16	0.878	15.16 ± 8.11
HKLS_17	0.878	14.72 ± 8.25
HKLS_18	0.876	15.48 ± 8.15
HKLS_19	0.875	15.63 ± 8.18
HKLS_20	0.875	15.57 ± 8.16
HKLS_21	0.876	15.22 ± 8.08
HKLS_22	0.876	15.52 ± 8.16

Results of the correlation matrix

**Figure 1:**
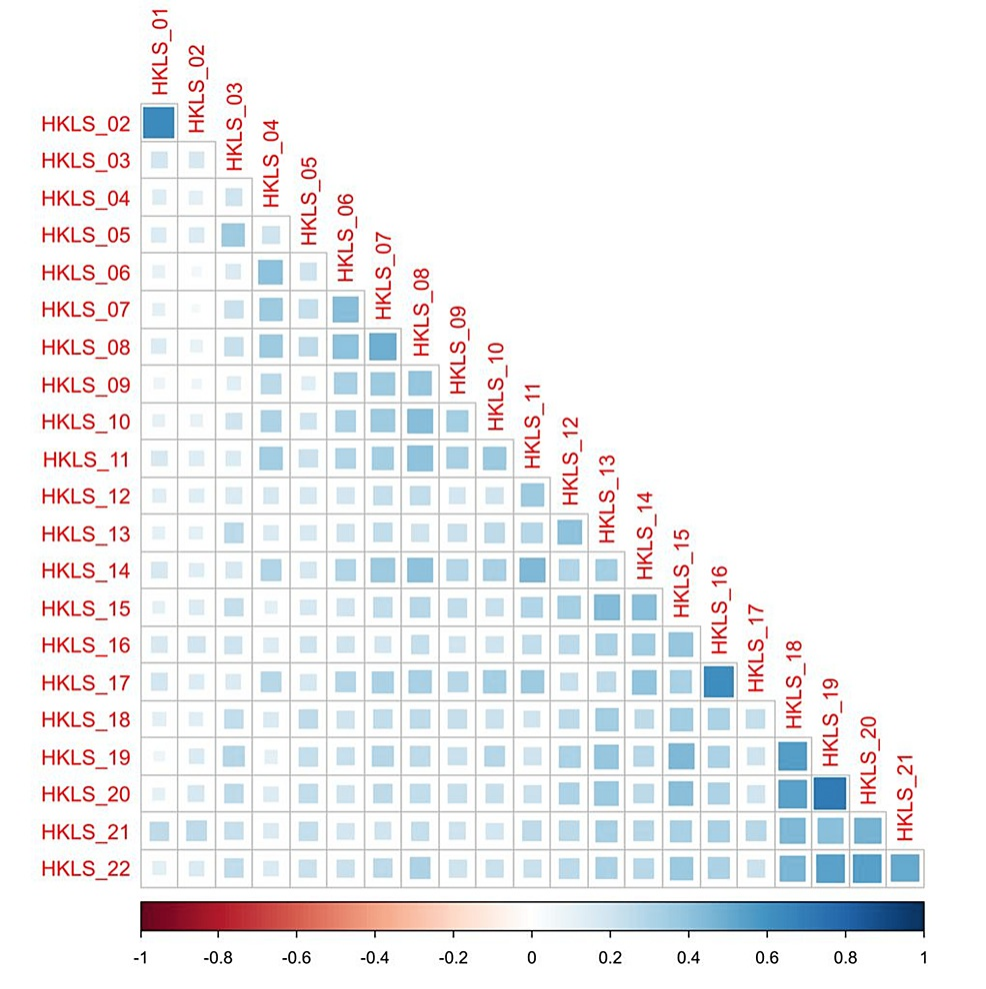
A correlogram depicting a correlation matrix for the association between the 22 items of the HK-LS questionnaire. HK-LS: Hypertension Knowledge-Level Scale.

Based on the results of the correlation analysis shown in Figure 1, all the items of the HK-LS questionnaire were significantly correlated with each other except a non-significant correlation between statements two and seven (r=0.05, p=0.091). All the correlations were positive. The strongest correlations were apparent between items 19 and 20 (r=0.70, p<0.0001), items one and two (r=0.64, p<0.0001), and items 18 and 19 (r=0.56, p<0.0001). Conversely, the weakest associations were reported among items two and six (r=0.06, p<0.05), items two and nine (r=0.07, p<0.05), and items one and 19 (r=0.07, p<0.05). More details about the correlation coefficients and the significance values are demonstrated in Appendix 2, Table 7.

Discriminant validity

Results of the discriminant validity showed that a personal history of hypertension was associated with significantly higher scores of two HK-LS subscales, including definition (1.21 ± 0.79 vs 0.96 ± 0.86, p<0.001) and complications (3.95 ± 1.57 vs 3.74 ± 1.62, p=0.004). However, the drug compliance score was significantly lower among participants with a personal history of hypertension compared to their peers (2.24 ± 1.41 vs 2.54 ± 1.27, p<0.001). Other subscales and the overall HK-LS score did not differ significantly based on the patient's personal history of hypertension. Therefore, the Arabic version of the survey was able to discriminate participants with hypertension for two subscales (out of six). Details of the differences in knowledge scores based on the personal history of hypertension are shown in Table 4. 

**Table 4:** Differences in knowledge scores based on the personal history of hypertension. *p<0.05.

Parameter	Hypertension (personal)		
	No, N=1049	Yes, N=370	p-value
Definition	0.96 ± 0.86	1.21 ± 0.79	<0.001*
Medical treatment	2.64 ± 1.26	2.67 ± 1.11	0.669
Drug compliance	2.54 ± 1.27	2.24 ± 1.41	<0.001*
Lifestyle	3.70 ± 1.50	3.84 ± 1.36	0.244
Diet	1.06 ± 0.92	1.15 ± 0.86	0.097
Complications	3.74 ± 1.62	3.95 ± 1.57	0.004*
Overall knowledge score	14.64 ± 5.15	15.07 ± 4.55	0.421

Regarding the family history of hypertension, the overall knowledge score was significantly higher among participants with a positive history of hypertension among family and/or friends (15.60 ± 4.44 vs 12.42 ± 5.69, p<0.001). Furthermore, the survey was able to discriminate the differences in knowledge among participants with a positive familial history of hypertension across different HK-LS subscales, including definition (1.07 ± 0.83 vs 0.90 ± 0.88, p=0.001), medical treatment (2.86 ± 1.10 vs 2.07 ± 1.34, p<0.001), drug compliance (2.62 ± 1.25 vs 2.04 ± 1.39, p<0.001), lifestyle (3.91 ± 1.32 vs 3.27 ± 1.71, p<0.001), diet (1.16 ± 0.90 vs 0.88 ± 0.91, p<0.001), and complications (3.99 ± 1.46 vs 3.25 ± 1.85, p<0.001). Table 5 presents the differences in knowledge scores based on the history of hypertension among family and/or friends.

**Table 5:** Differences in knowledge scores based on the history of hypertension among family and/or friends. *p<0.05.

Parameter	History of hypertension among family and/or friends		
	No, N=379	Yes, N=1,040	p-value
Definition	0.90 ± 0.88	1.07 ± 0.83	0.001*
Medical treatment	2.07 ± 1.34	2.86 ± 1.10	<0.001*
Drug compliance	2.04 ± 1.39	2.62 ± 1.25	<0.001*
Lifestyle	3.27 ± 1.71	3.91 ± 1.32	<0.001*
Diet	0.88 ± 0.91	1.16 ± 0.90	<0.001*
Complications	3.25 ± 1.85	3.99 ± 1.46	<0.001*
Overall knowledge score	12.42 ± 5.69	15.60 ± 4.44	<0.001*

## Discussion

Several studies have focused on testing the validity and reliability of the HK-LS scale in regions such as Europe, where various languages are spoken [7-11]. This study aimed to construct the validity and reliability of the Arabic version of the Hypertension Knowledge-Level Scale (HK-LS) among the Saudi population. Our results support the findings from a study conducted in Jordan using the same scale [1]. We found that the Arabic version has a Cronbach's alpha coefficient range of 0.883, which indicates that an Arabic version is a reliable tool for measuring HK-LS in Saudi Arabia. However, our result showed that Cronbach's alpha coefficient for each subscale demonstrated that the lowest values refer to "medical treatment."

Regarding "medical treatment," many participants know the correct answer that they should take it daily. Still, they don't do it, which can be demonstrated when we observe that the result of drug compliance in patients with a history of hypertension is significantly lower than that of their peers. This finding was consistent in other literature that explained apparent conflicting answers in opposing propositions; the statement "medicines for high blood pressure should be taken daily" is different from the statement "people with high blood pressure should only take their medications when they feel bad." Nevertheless, several individuals proclaimed that both were correct [11]. This response could justify the low value of Cronbach's alpha.

Considering the reliability and internal consistency of the HK-LS subscales, Cronbach's alpha coefficient was also low for diet subscales (Cronbach's alpha=0.723). The complication subscale showed the highest internal consistency indicators (Cronbach's alpha=0.846) and the definition subscale (Cronbach's alpha=0.789). The Cronbach's alpha of the overall HK-LS was 0.883, indicating a reliable tool. This finding is higher when compared to the reliability of the Brazilian version of HK-LS, where the overall coefficient was 0.74 [11]. Similarly, another study reported an overall Cronbach alpha coefficient of 0.82 for the entire scale, while subscale coefficients were 0.92, 0.59, 0.67, 0.77, 0.72, and 0.76 for the definition, medical treatment, drug compliance, lifestyle, diet, and complications, respectively [5].

The statistical analysis also showed that the removal of the statement "increased diastolic blood pressure also indicates increased blood pressure" and the removal of the statement "high diastolic or systolic blood pressure indicates increased blood pressure," both statements at part "definition," would increase Cronbach's alpha=0.885 instead of Cronbach's alpha=0.883. The findings could be due to the difficulty of understanding the statements. 

Regarding the personal history of hypertension, our results showed significantly higher scores of two HK-LS subscales, including definitions and complications, among hypertensive participants. This finding can be explained by their knowledge of the disease and frequent follow-up visits to raise their awareness regarding complications. This finding was consistent with another study that reported that knowledge was significantly higher among hypertensive participants than among non-hypertensive participants [1]. An Indonesian study found a similar result, where the discriminative validity test revealed that the HK-LS scores between the two groups of participants involved were statistically significant [9]. In another similar study, the knowledge level score of individuals with cardiovascular diseases or family history was significantly greater than that of people without cardiovascular diseases or family history [14]. However, our findings showed that the drug compliance score was significantly lower among participants with a history of hypertension compared to their peers. This finding may be attributed to the nature of the disease, as hypertension is a silent disease. Therefore, most patients take the medication when they only start to have symptoms. This result was consistent with other literature that revealed a significant correlation between medication adherence and illness perception [15-17]. Moreover, a newly published study in Saudi Arabia showed poor adherence to hypertension medication among the Saudi population [18]. 

Regarding the results of the correlation analysis, all the items were observed to be significantly correlated with each other, except for a non-significant correlation between statements two and seven (r=0.05, p=0.091). All the correlations were positive. In the correlation matrix of the Brazilian study, the values ranged from −0.181 to +0.540, with 196 correlations being positive and 35 being negative [11]. In the Turkish scale, a significant correlation was observed between the knowledge score and demographic variables such as age, gender, educational level, and personal history of hypertension. No correlation was observed between knowledge score and having a job [5]. In addition, the overall knowledge score and a positive family and/or friends history of hypertension were correlated (p<0.001). In contrast, a previous Greek study reported no observed correlation between knowledge level and age, gender, and education level [7]. 

A strength of this study is its use of widely used instruments to measure HK-LS among Arabic-speaking patients, which is valuable and sets the stage for utilizing this important tool for measuring HK-LS among populations in numerous countries in the Gulf region and the Middle East where the Arabic language is widely spoken. However, the study had some limitations. As with questionnaire-based studies, there could be a reporting bias which had influenced the responses received from the participant to under- or overestimate their knowledge. Also, the difficulty of understanding for some participants was considered a limitation of this study, as the respective capacity for interpretation may interfere with the application of the instrument. 

Based on the previously mentioned findings, we recommend that the authorities and stakeholders take responsibility for constructing an agreed-upon valid Arabic version of the different international medical scales. Also, collaboration with Arab researchers from various Arab countries is recommended for more generalizable results and achievements. 

## Conclusions

The Arabic version of the HK-LS is a valid and reliable tool for measuring knowledge about hypertension in Saudi Arabia. Its testing is curial to enable this instrument's use in settings including community health clinics and hospitals. In addition, patients are encouraged to actively raise their knowledge about hypertension and its health consequences by developing educational programs and campaigns to increase public awareness regarding hypertension.

## Appendices

Appendix 1

**Table 6:** The correct responses of HK-LS items. HK-LS: Hypertension Knowledge-Level Scale.

Subscale	Item#	Description	Correct answer
Definition	HKLS_01	Increased diastolic blood pressure also indicates increased blood pressure	True
	HKLS_02	High diastolic or systolic blood pressure indicates increased blood pressure	True
Medical treatment	HKLS_03	Drugs for increased blood pressure must be taken everyday	True
	HKLS_04	Individuals with increased blood pressure must take their medication only when they feel ill	False
	HKLS_05	Individuals with increased blood pressure must take their medication throughout their life	True
	HKLS_06	Individuals with increased blood pressure must take their medication in a manner that makes them feel good	False
Drug compliance	HKLS_07	If the medication for increased blood pressure can control blood pressure, there is no need to change lifestyles	False
	HKLS_08	Increased blood pressure is the result of aging, so treatment is unnecessary	False
	HKLS_09	If individuals with increased blood pressure change their lifestyles, there is no need for treatment	False
	HKLS_10	Individuals with increased blood pressure can eat salty foods as long as they take their drugs regularly	False
Lifestyle	HKLS_11	Individuals with increased blood pressure can drink alcoholic beverages	False
	HKLS_12	Individuals with increased blood pressure must not smoke	True
	HKLS_13	Individuals with increased blood pressure must eat fruits and vegetables frequently	True
	HKLS_14	For individuals with increased blood pressure, the best cooking method is frying	False
	HKLS_15	For individuals with increased blood pressure, the best cooking method is boiling or grilling	True
Diet	HKLS_16	The best type of meat for individuals with increased blood pressure is white meat	True
	HKLS_17	The best type of meat for individuals with increased blood pressure is red meat	False
Complications	HKLS_18	Increased blood pressure can cause premature death if left untreated	True
	HKLS_19	Increased blood pressure can cause heart diseases, such as heart attack, if left untreated	True
	HKLS_20	Increased blood pressure can cause strokes, if left untreated	True
	HKLS_21	Increased blood pressure can cause kidney failure, if left untreated	True
	HKLS_22	Increased blood pressure can cause visual disturbances, if left untreated	True

Appendix 2

**Table 7:** Results of the Spearman’s correlation analysis for the association between the items of the HK-LS questionnaire. HK-LS: Hypertension Knowledge-Level Scale. ***p<0.0001, **p<0.01, and *p<0.05.

	HKLS_01	HKLS_02	HKLS_03	HKLS_04	HKLS_05	HKLS_06	HKLS_07	HKLS_08	HKLS_09	HKLS_10	HKLS_11	HKLS_12	HKLS_13	HKLS_14	HKLS_15	HKLS_16	HKLS_17	HKLS_18	HKLS_19	HKLS_20	HKLS_21	HKLS_22
HKLS_01	1.00																					
HKLS_02	0.64***	1.00																				
HKLS_03	0.18***	0.17***	1.00																			
HKLS_04	0.14***	0.12***	0.20***	1.00																		
HKLS_05	0.15***	0.16***	0.35***	0.20***	1.00																	
HKLS_06	0.09**	0.06*	0.15***	0.40***	0.20***	1.00																
HKLS_07	0.11***	0.05	0.22***	0.35***	0.24***	0.42***	1.00															
HKLS_08	0.15***	0.10**	0.22***	0.36***	0.26***	0.40***	0.49***	1.00														
HKLS_09	0.08**	0.07*	0.13***	0.26***	0.13***	0.33***	0.36***	0.39***	1.00													
HKLS_10	0.10**	0.10**	0.20***	0.30***	0.20***	0.31***	0.36***	0.42***	0.33***	1.00												
HKLS_11	0.16***	0.14***	0.14***	0.34***	0.22***	0.29***	0.34***	0.41***	0.32***	0.37***	1.00											
HKLS_12	0.13***	0.14***	0.16***	0.17***	0.18***	0.18***	0.23***	0.24***	0.19***	0.20***	0.35***	1.00										
HKLS_13	0.11***	0.12***	0.26***	0.15***	0.16***	0.21***	0.24***	0.20***	0.22***	0.25***	0.29***	0.40***	1.00									
HKLS_14	0.17***	0.14***	0.16***	0.30***	0.17***	0.29***	0.36***	0.40***	0.28***	0.32***	0.44***	0.29***	0.34***	1.00								
HKLS_15	0.11***	0.14***	0.23***	0.12***	0.17***	0.19***	0.25***	0.27***	0.24***	0.23***	0.29***	0.34***	0.44***	0.41***	1.00							
HKLS_16	0.17***	0.19***	0.21***	0.15***	0.20***	0.20***	0.19***	0.23***	0.20***	0.20***	0.25***	0.27***	0.32***	0.35***	0.38***	1.00						
HKLS_17	0.18***	0.15***	0.15***	0.28***	0.18***	0.29***	0.32***	0.32***	0.28***	0.33***	0.36***	0.23***	0.26***	0.40***	0.33***	0.61***	1.00					
HKLS_18	0.12***	0.13***	0.25***	0.15***	0.25***	0.20***	0.24***	0.25***	0.24***	0.22***	0.20***	0.27***	0.35***	0.25***	0.35***	0.32***	0.24***	1.00				
HKLS_19	0.07*	0.16***	0.29***	0.12***	0.24***	0.23***	0.29***	0.25***	0.22***	0.28***	0.21***	0.32***	0.39***	0.28***	0.45***	0.31***	0.21***	0.56***	1.00			
HKLS_20	0.11***	0.18***	0.26***	0.14***	0.25***	0.21***	0.25***	0.25***	0.22***	0.23***	0.21***	0.30***	0.37***	0.26***	0.42***	0.31***	0.20***	0.54***	0.70***	1.00		
HKLS_21	0.25***	0.26***	0.22***	0.15***	0.24***	0.19***	0.20***	0.24***	0.20***	0.18***	0.26***	0.27***	0.32***	0.29***	0.33***	0.32***	0.28***	0.45***	0.42***	0.46***	1.00	
HKLS_22	0.11***	0.16***	0.25***	0.15***	0.21***	0.22***	0.26***	0.30***	0.21***	0.21***	0.21***	0.26***	0.31***	0.27***	0.36***	0.32***	0.21***	0.45***	0.54***	0.55***	0.51***	1.00

## References

[1] Eshah NF, Al-Daken LI (2016). Assessing public's knowledge about hypertension in a community-dwelling sample. J Cardiovasc Nurs.

[2] Vancini-Campanharo CR, Oliveira GN, Andrade TF, Okuno MF, Lopes MC, Batista RE (2015). Systemic arterial hypertension in the emergency service: medication adherence and understanding of this disease. Rev Lat Am Enfermagem.

[3] Malachias MV, Gomes MA, Nobre F, Alessi A, Feitosa AD, Coelho EB (2016). 7th Brazilian guideline of arterial hypertension: chapter 2-diagnosis and classification. Arq Bras Cardiol.

[4] Mantovani MDF, Arthur JP, Mattei ÂT, Ulbrich EM, Kalinke LP (2015). Protocolos clínicos na orientação de pessoas com doença crônica. Cogitare Enferm.

[5] Erkoc SB, Isikli B, Metintas S, Kalyoncu C (2012). Hypertension Knowledge-Level Scale (HK-LS): a study on development, validity and reliability. Int J Environ Res Public Health.

[6] Zinat Motlagh SF, Chaman R, Ghafari SR, Parisay Z, Golabi MR, Eslami AA, Babouei A (2015). Knowledge, treatment, control, and risk factors for hypertension among adults in southern Iran. Int J Hypertens.

[7] Chatziefstratiou AA, Giakoumidakis K, Fotos NV, Baltopoulos G, Brokalaki-Pananoudaki H (2015). Translation and validation of the Greek version of the hypertension knowledge-level scale. J Clin Nurs.

[8] Jankowska-Polańska B, Uchmanowicz I, Dudek K, Mazur G (2016). Relationship between patients' knowledge and medication adherence among patients with hypertension. Patient Prefer Adherence.

[9] Ernawati I, Fandinata SS, Permatasari SN (2020). Translation and validation of the Indonesian version of the hypertension knowledge-level scale. Open Access Maced J Med Sci.

[10] Arthur JP, Mantovani MF, Ferraz MI, Mattei ÂT, Kalinke LP, Corpolato RC (2018). Translation and cross-cultural adaptation of the Hypertension Knowledge-Level Scale for use in Brazil. Rev Lat Am Enfermagem.

[11] Hereibi MJ, Arthur JP, Mantovani MF, Mattei ÂT, Viante WJ, Bortolato-Major C (2021). Construct validity and reliability of the Brazilian version of Hypertension Knowledge-Level Scale. Rev Gaucha Enferm.

[12] Nadeem MK, Mari A, Iftikhar S, Khatri A, Sarwar T, Patel MJ (2019). Hypertension-related knowledge and its relationship with blood pressure control in hypertensive patients visiting a semi-private tertiary-care charity hospital in Karachi, Pakistan. Cureus.

[13] Tsang S, Royse CF, Terkawi AS (2017). Guidelines for developing, translating, and validating a questionnaire in perioperative and pain medicine. Saudi J Anaesth.

[14] Arikan I, Metintaş S, Kalyoncu C, Yildiz Z (2009). The cardiovascular disease risk factors knowledge level (CARRF-KL) scale: a validity and reliability study. Turk Kardiyol Dern Ars.

[15] Shakya R, Shrestha S, Gautam R (2020). Perceived illness and treatment adherence to hypertension among patients attending a tertiary hospital in Kathmandu, Nepal. Patient Prefer Adherence.

[16] Rajpura JR, Nayak R (2014). Role of illness perceptions and medication beliefs on medication compliance of elderly hypertensive cohorts. J Pharm Pract.

[17] Hsiao CY, Chang C, Chen CD (2012). An investigation on illness perception and adherence among hypertensive patients. Kaohsiung J Med Sci.

[18] Thirunavukkarasu A, Naser Abdullah Alshahrani A, Mazen Abdel-Salam D (2022). Medication adherence among hypertensive patients attending different primary health centers in Abha, Saudi Arabia: a cross-sectional study. Patient Prefer Adherence.

